# Transcriptomic analysis of the honey bee (*Apis mellifera*) queen spermathecae reveals genes that may be involved in sperm storage after mating

**DOI:** 10.1371/journal.pone.0244648

**Published:** 2021-01-08

**Authors:** Juliana Rangel, Tonya F. Shepherd, Alejandra N. Gonzalez, Andrew Hillhouse, Kranti Konganti, Nancy H. Ing

**Affiliations:** 1 Department of Entomology, Texas A&M University, College Station, Texas, United States of America; 2 Texas A&M Institute of Genome Sciences and Society, Texas A&M University, College Station, Texas, United States of America; 3 Department of Animal Science, Texas A&M University, College Station, Texas, United States of America; University of Alberta, CANADA

## Abstract

Honey bee (*Apis mellifera*) queens have a remarkable organ, the spermatheca, which successfully stores sperm for years after a virgin queen mates. This study uniquely characterized and quantified the transcriptomes of the spermathecae from mated and virgin honey bee queens via RNA sequencing to identify differences in mRNA levels based on a queen’s mating status. The transcriptome of drone semen was analyzed for comparison. Samples from three individual bees were independently analyzed for mated queen spermathecae and virgin queen spermathecae, and three pools of semen from ten drones each were collected from three separate colonies. In total, the expression of 11,233 genes was identified in mated queen spermathecae, 10,521 in virgin queen spermathecae, and 10,407 in drone semen. Using a cutoff log_2_ fold-change value of 2.0, we identified 212 differentially expressed genes between mated and virgin spermathecal queen tissues: 129 (1.4% of total) were up-regulated and 83 (0.9% of total) were down-regulated in mated queen spermathecae. Three genes in mated queen spermathecae, three genes in virgin queen spermathecae and four genes in drone semen that were more highly expressed in those tissues from the RNA sequencing data were further validated by real time quantitative PCR. Among others, expression of Kielin/chordin-like and Trehalase mRNAs was highest in the spermathecae of mated queens compared to virgin queen spermathecae and drone semen. Expression of the mRNA encoding Alpha glucosidase 2 was higher in the spermathecae of virgin queens. Finally, expression of Facilitated trehalose transporter 1 mRNA was greatest in drone semen. This is the first characterization of gene expression in the spermathecae of honey bee queens revealing the alterations in mRNA levels within them after mating. Future studies will extend to other reproductive tissues with the purpose of relating levels of specific mRNAs to the functional competence of honey bee queens and the colonies they head.

## 1. Introduction

In insects, success during sexual reproduction is achieved by the implementation of species- and sex-specific strategies that involve direct contact between the male’s sperm and seminal fluid and the female’s eggs and the epithelial cells along the female’s reproductive tract. These interactions can affect mating behavior, gamete production, fertilization efficiency, sperm competition and sperm storage [1,2]. In particular, sperm storage, the maintenance of sperm inside a female’s reproductive tract for a sustained period of time, is a key feature of reproductive success in many insects [3,4]. For example, the myriad of sperm storage organs that have evolved in fruit fly species in the genus *Drosophila* [5] have been optimized such that one male’s seminal fluid and accessory gland proteins increase the survival of all other males’ ejaculates collected by a female during mating [6,7]. In another dipteran species, the mosquito *Aedes aegypti*, females mate with only one male but store their mate’s sperm for a long period of time [8,9]. A recent RNA sequencing study of the spermatheca of virgin and mated *A*. *aegypti* females found eight spermatheca-specific transcripts that are responsible for the nourishment, maintenance and protection of male sperm [4].

Long-term sperm storage by reproductive females is also common in eusocial insects including ants, wasps and some bees, whereby queens typically mate early in life and store the sperm received during mating for years [10–12]. The sperm is stored in the spermatheca, allowing mating and fertilization to occur asynchronously [3]. Several factors in the female’s spermathecal fluids and the male’s seminal secretions have proven to be crucial for long-term storage and mating success in eusocial insects. In the ant *Atta colombica*, secretions from the male accessory glands greatly increase sperm viability upon ejaculation inside the female [13]. In *Acromyrmex echinatior* ants, sperm have a “self-non-self” recognition system in which sperm motility is lower in the presence of a male’s own seminal fluids, but it is higher when mixed with the seminal fluids of other males’ sperm, suggesting that enhanced sperm motility is costly and thus is only implemented as needed during competition between males [14].

In honey bees (*Apis mellifera*), a queen’s genitalia and spermatheca are located in the lower section of the reproductive tract, where sperm storage, maintenance, release and fertilization occur [1,15]. Virgin honey bee queens mate with an average of 12 drones (range = 1–28) in quick succession during one or multiple mating flights [16,17]. After copulation, less than 10% of the semen remains inside the queen [18] and only about 3% of the sperm actively migrates to the spermatheca for long-term storage [12]. Interestingly, live sperm can “drag” dead sperm cells to the spermatheca [19], increasing the overall number of sperm cells that reach the spermatheca upon mating. The spermatheca of a newly mated queen can contain between four and seven million sperm cells [20–22], which she uses to fertilize 1,000 or more eggs daily for the production of worker offspring over her two- to three-year lifespan [23].

Given that honey bee queens need to store the sperm received from their mates for such an extended period of time, long-term viability of the males’ ejaculates is crucial to male reproductive success [24–26]. A combination of factors including sugars, pH, ions, high expression of antioxidative enzymes, as well as the presence of proteins involved in energy and metabolism in the male’s seminal fluid, are essential for sperm viability and longevity before and during storage [27–41].

Despite the important role played by sperm storage in honey bee reproduction, the molecular mechanisms by which sperm remain viable for years inside the queen’s spermatheca are not completely understood. Proteomic studies have identified numerous proteins in the queen spermatheca [34] and drone seminal fluid [35] and have shown changes in proteome composition between recently ejaculated sperm and sperm stored in the queen’s spermatheca [38]. However, the molecular basis of sperm longevity within the spermatheca cannot be clearly elucidated via protein expression because many of the protein profiles from such studies may be from highly expressed proteins produced by other tissues and would thus require simultaneous comparative experiments between the spermatheca and other organs. A more informative approach, such as that taken in our study, is to elucidate large-scale gene expression upon mating in honey bee queens through next generation, high-throughput RNA sequencing of the spermathecal organ before and after mating.

Some of the behavioral and physiological changes known to occur in honey bee queens after copulation [42–44] are likely associated with changes in gene expression based on the queen’s mating status [30,41,45]. With this in mind, we searched for candidate genes that may contribute to sustained sperm longevity in queens by performing differential gene expression analyses between the spermathecae of virgin queens and newly mated queens, and compared them to that of drone semen, paying particular attention to gene expression patterns that were unique to each tissue type. We discuss the putative functions of some gene products that are induced in the spermatheca after mating and thus may be involved in sperm storage. Our approach represents, to our knowledge, the first transcriptomic study of the honey bee queen spermatheca that focuses on the genes that may be involved in long-term sperm storage after mating, providing a foundation for the development of genetic markers of reproductive quality in honey bee queens and drones. Results from future studies using the honey bee as a model organism can help assess the reproductive health of other important pollinator species.

## 2. Materials and methods

### 2.1. Source of bees

We obtained three virgin queens and three recently mated queens that were shipped by a commercial queen producer located in California’s Central Valley (Olivarez Honey Bees, Inc., Orlando, CA). The virgin queens were approximately two weeks old and the mated queens were approximately three to four weeks old. All queens were obtained from the same Carniolan stock source colony and thus were sisters to each other. The queens were individually labeled, caged in a box containing worker attendants and food, and shipped to our laboratory facility at Texas A&M University in College Station, TX. The queens were kept at room temperature until they were dissected for collection of the spermatheca. Sexually mature drones (n = 3 biological replicates, using a pool of ten drones per biological replicate), approximately 15 to 18 days post emergence, were also collected for dissection and semen collection from each of three colonies located at the Janice and John G. Thomas Honey Bee Facility of Texas A&M University’s RELLIS Campus in Bryan, TX.

### 2.2. Sample and tissue collection

**Fig 1:**
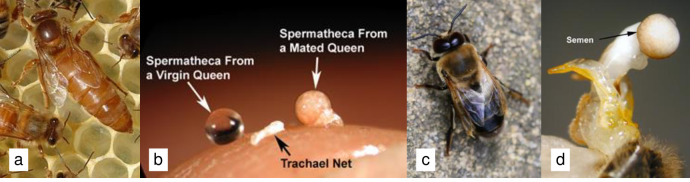
Pictures depicting a) a honey bee queen; b) a virgin queen’s spermatheca with the tracheal net removed (left), a tracheal net (middle) and a mated queen’s spermatheca (diameter = ∼1 mm) filled with semen and the tracheal net removed (right) on a fingertip; c) an adult drone; and d) a fully everted endophallus with semen expressed at the end of the bulb (diameter of the semen droplet = ∼0.5 mm). Photos credits: a and c) Pixabay.com; b and d) Sue Cobey.

Each queen was immobilized by placing her in a -20^°^C freezer for 3 to 5 min. Once immobilized, she was decapitated on a bed of dry ice to keep all tissues suitable for RNA extraction. We then dissected the queen’s spermatheca from the abdomen, removing the tracheal net covering the spermatheca with forceps (Fig 1A and 1B). The spermatheca was pressed against the wall of a microcentrifuge tube to release its contents inside the tube after adding 50 μL PureZOL™ RNA isolation reagent (Bio-Rad Laboratories, Inc., Hercules, CA). Each sample was frozen individually at -80^°^C until it was used for RNA extraction (see below). The spermathecal samples from both virgin and mated queens were handled in the same manner by the same person to control for variation due to handling. Drones were decapitated in a similar manner as queens and pressure was then applied to their abdomen to evert the endophallus. A micropipette was used to collect the semen laying on top of a bed of mucus at the tip of the bulb (Fig 1C and 1D), as done previously [41]. We pooled the semen from ten drones from each of the three colonies. Therefore, each biological replicate contained the semen from ten drones from the same colony. We added 50 μL PureZOL™ RNA isolation reagent (Bio-Rad Laboratories, Inc., Hercules, CA) and then froze the three pooled semen samples at -80^°^C until they were used for RNA extraction (see below).

### 2.3. RNA extraction and cDNA library synthesis

We purified total RNA from three biological replicates per tissue type: the spermatheca from three virgin queens, the spermatheca from three mated queens, and three pooled drone semen samples (n = 3 drones per replicate), which was considered an acceptable number of replicates for these type of studies in 2017, the year when the analysis was conducted [46]. Each sample was individually placed in 1.5 mL microtubes with 50 μL PureZOL™ RNA isolation reagent (Bio-Rad Laboratories, Inc., Hercules, CA) and the tissues therein were ground with a pellet mixer (VWR^®^ catalog number 47747–307, Radnor, PA). RNA from each sample was extracted using the standard protocol for PureZOL™. After adding 1 μL of 20 μg/μL RNAse-free glycogen, RNA was precipitated overnight at -20°C. RNA pellets were created through 10 min of centrifugation at 12,000 x *g* in a microcentrifuge kept at 4°C. The pellets were washed in 75% ethanol, air dried for 10 min and resuspended in 25 μL of nuclease-free water. RNA concentrations were measured in a Qubit®2.0 fluorometer with a Qubit® RNA HS Assay Kit (Life Technologies Corporation, Grand Island, NY).

Fifty ng aliquots of each RNA sample were sent to the Texas A&M University’s Institute for Genome Sciences and Society Core (College Station, TX) for quality assessment (done on a BioAnalyzer 2100), cDNA library construction and sequencing. The cDNA libraries used for sequencing were prepared according to the NuGEN Technologies (Tecan Genomics, Inc., Redwood City, CA) protocol using the Ovation SoLo RNA-Seq system. After adapter ligation, ribosomal transcripts were depleted using a custom insert dependent adapter cleavage (InDA-C) probe pool from NuGEN Technologies (Tecan Genomics, Inc., Redwood City, CA) designed for ribosomal RNA sequences in the *A*. *mellifera* genome.

### 2.4. RNA sequencing and bioinformatics analyses for differential gene expression

Libraries, in equimolar concentrations, were sequenced on an Illumina NextSeq 500, with a high-output 150 base-pair sequencing run, using the manufacturer’s supplied custom sequencing read 1 primer on four sequencing lanes. After sequencing, the first five bases were trimmed as suggested in the Ovation Solo RNA-seq protocol (https://www.nugen.com/sites/default/files/M01406_v4_User_Guide%3A_Ovation_SoLo_RNA-Seq_System_1287.pdf). A total of 424 million reads were checked to trim any adapter sequences and low-quality bases using Trimmomatic [47]. Read mapping was performed using HISAT version 2.0.5 [48]. The genome Amel_4.5 from NCBI was used for contig assembly (https://www.ncbi.nlm.nih.gov/assembly/GCF_000002195.4/). HTSeq [49] was used to generate raw read counts per gene using an intersection-nonempty parameter to account for ambiguous read mappings.

### 2.5. Functional clustering analysis of differentially expressed genes

Tables of functional clustering of differentially expressed genes were generated using the Database for Annotation, Visualization and Integrated Discovery (D.A.V.I.D.) platform [50,51] (https://david.ncifcrf.gov/home.jsp). Gene lists from differential expression analysis of the RNA sequencing data were filtered to isolate genes that were different by either ≥ 4.0-fold (up-regulated) or ≤ -4.0-fold (down-regulated). The gene lists were uploaded to D.A.V.I.D. and referenced against the in-house database for *A*. *mellifera* on BeeBase. The “Functional Annotation Clustering” under “lowest” classification stringency was used to study the gene list. We report all terms with FDR cutoff values ≤ 0.05.

### 2.6. Confirmation of differential expression of selected gene products by RT-qPCR

From the RNA sequencing data, ten mRNA targets were chosen to be analyzed for tissue specificity using RT-qPCR (S1 Table). The mRNAs chosen for RNA-seq confirmation were selected because they were the most prevalent (based on the sequencing data) in each tissue type. The mRNAs chosen for their high level of expression in the spermathecae of mated queens were GB54516 (Uncharacterized protein), GB53925 (Kielin/chordin-like) and GB43575 (Trehalase). The mRNAs selected for their expression in the spermathecae of virgin queens were GB43248 (α Glucosidase 2), GB44112 (Melittin) and GB54549 (α Glucosidase 1). The drone semen-specific mRNAs chosen were GB48478 (Multiple inositol polyphosphate phosphatase 1), GB54806 (Facilitated trehalose transporter 1), GB45850 (Clavesin2) and GB40598 (Bumetanide-sensitive Na(K)Cl cotransporter). The mRNA chosen for normalizing the data was GB41358 (encoding Elongation factor 1-alpha F2, or EF1aF2) because of its high and invariant expression across samples in the RNA sequencing data, as well as having previously been validated as a reference gene for gene expression studies of honey bee tissues [52].

Primers were designed with the Primer-BLAST software to span exon junctions and to detect all isoforms for each gene product of interest [53]. All primer sequences are listed in S1 Table. The RNA samples were used to synthesize cDNA for qPCR in a 20 μL volume. For each sample, 50 ng of total RNA from the same tissues was used for RNA sequencing. Using the same source material for RNA sequencing, RT-qPCR was done to reduce the amount of environmental variability surrounding the rearing of drones and queens in different sample types. The total RNA from each sample was treated with DNase and reverse transcribed using the iScript^TM^ gDNA Clear cDNA Synthesis kit (Bio-Rad Laboratories, Inc.). Synthesized cDNA was diluted by four-fold and 1 μL of that dilution was used in a 10 μL qPCR reaction. Diluted cDNA acted as template for all qPCRs. All amplifications used Power SYBR^TM^ Green (Thermo Fisher Scientific, Waltham, MA) in triplicate reactions (10 μL) with primers at 80 nM final concentrations. Standard cycling conditions (50°C for 2 min, 95°C for 2 min, then 40 cycles of 95°C for 15 s and 60°C for 1 min) and melt curve analyses (65°C to 95°C in 0.5°C increments every 5 s) were used on a CFX^TM^ Real-Time system (Bio-Rad Laboratories, Inc.). All qPCR analyses were done using the CFX Manager 3.1 software.

Amplification efficiencies were calculated and used for correction in all normalized fold expression analyses, performed by the CFX^TM^ Manager 3.1 software. M values for the normalizer were < 0.5 for all runs, as calculated by CFX Manager 3.1. Linear values (2^-ΔΔC_t_) are reported as means and standard error of the mean (SEM) for each tissue [54]. The RT-qPCR data are reported after setting the lowest value for that gene/tissue at one and reporting up-regulation of that gene in the other two tissues as fold change values.

### 2.7. Statistical analysis

For the RNA sequencing data, the DESeq2 program used Negative Binomial Distribution to estimate means and variances for each gene per sample based on library size and then performs Wald test to extract *P* and FDR values [55]. If the latter was < 0.05, genes were considered differentially expressed. For the RT-qPCR data, we performed one-way ANOVA tests to determine if there were statistical differences in gene expression values between the spermathecae of virgin queens, the spermathecae of mated queens and drone semen. In cases where we found statistical differences in gene expression among queen types, or between queens and drones, we conducted pairwise Student’s *t* tests to discern differences between tissue types. All tests were performed using the statistical software JMP^®^ 12.0 (SAS Inc., Cary, NC). We set the level of statistical significance at *P* < 0.05 for all tests and report all descriptive statistics as means ± SEM.

## 3. Results

### 3.1. RNA yield and purity

RNA purified from individual mated queen spermatheca had average yields of 639 ± 264 ng. Similarly, RNA yields from each virgin queen spermatheca averaged 650 ± 315 ng. However, preparations from pooled semen from ten drones from the same colony (per biological replicate) yielded less RNA, with an average of only 92 ± 17 ng. RNA purity was assessed as described in Gonzalez et al. [41].

### 3.2. RNA sequencing of three honey bee tissues

RNA sequencing data were generated from three honey bee tissues: mated queen spermatheca, virgin queen spermatheca, and drone semen. A total of nine cDNA libraries were made, one for each of three biological replicates for each tissue. RNA sequencing of all libraries resulted in approximately 393 million high-quality reads (92.7% of the total 424 million reads). Of these, 39 million reads were further filtered out. The remaining 385 million reads mapped to the *A*. *mellifera* genome. The number of total reads, mapped reads and corresponding genes are listed in S2 Table for each of the nine libraries. In total, the expression of 11,233 genes was identified in mated queen spermathecae, 10,521 in virgin spermathecae, and 10,407 in drone semen (not shown in S2 Table).

Because these data are novel, it is important to take note of the genes that were highly expressed in each tissue. The top 20 most prevalent genes by normalized level of expression in each tissue type are listed in Table 1. The complete lists of genes expressed in each tissue are presented in the S3 Table. The entire datasets for each library are available at NCBI (Bioproject # PRJNA542364).

**Table 1:** The top 20 most prevalent RNAs discovered in each honey bee tissue (i.e., mated queen spermatheca, virgin queen spermatheca, and drone semen) are listed along with their BeeBase gene identifiers (“Gene_ID”).

Gene_ID	Protein description	Average value
***Top 20 genes in mated queen spermatheca***		
*** ***	*** ***	*** ***
GB54516	Uncharacterized LOC100577150	3.37
GB49544	Vitellogenin	2.32
***GB43575***	***Trehalase***	2.11
GB47379	Histidine decarboxylase	1.96
GB46537	Deoxyribonuclease-2-alpha	1.95
***GB53925***	***Uncharacterized LOC724993***	1.92
GB52320	Uncharacterized LOC100578903	1.81
GB54454	Uncharacterized LOC724783	1.79
GB51306	Apidaecins type 73	1.79
GB43576	Trehalase-like	1.71
GB54517	Trypsin 3A1	1.70
GB49865	Protein drumstick	1.69
GB48134	L-lactate dehydrogenase-like	1.65
GB44493	Arylsulfatase B-like	1.65
GB50571	Uncharacterized LOC102655899	1.64
GB45906	Uncharacterized LOC410087	1.63
GB43711	Membralin-like	1.63
GB50574	Uncharacterized LOC102656231	1.59
GB50572	Uncharacterized LOC408538	1.58
GB47546	Apidaecin 1	1.57
***Top 20 genes in virgin queen spermetheca***		
***GB44112***	***Melittin***	4.08
GB44367	Phospholipase A2-like	3.56
***GB54549***	***Alpha glucosidase 1***	3.30
GB53887	Uncharacterized LOC100578389	2.95
GB52317	Secapin	2.85
GB53978	Early nodulin-75-like	2.72
***GB43248***	***Alpha glucosidase 2***	2.56
GB44100	Vegetative cell wall protein gp1-like	2.50
GB40136	Transmembrane protease serine 11B-like protein	2.33
GB53911	Peritrophin-1-like	2.16
GB40137	Transmembrane protease serine 11B-like protein	2.15
GB42434	Chitinase-3-like protein 1	2.12
GB44295	Sodium-dependent neutral amino acid transporter B(0)AT3	2.07
GB46286	Zinc carboxypeptidase-like	2.03
GB52161	Cuticular protein 28	2.02
GB50477	Uncharacterized LOC100577527	2.00
GB50761	Chymotrypsin-1	1.99
GB46587	Probable salivary secreted peptide	1.93
GB52318	Uncharacterized LOC102655788	1.92
GB50883	H/ACA ribonucleoprotein complex subunit 1-like	1.90
***Top 20 genes in drone semen***		
***GB48478***	***Multiple inositol polyphosphate phosphatase 1***	6.17
GB55813	Uncharacterized LOC100577773	5.73
GB41857	CRISP/Allergen/PR-1-like	4.63
GB54417	Farnesol dehydrogenase-like	4.05
***GB40598***	***Bumetanide-sensitive sodium-(potassium)-chloride cotransporter***	3.84
***GB45850***	***Clavesin-2***	3.66
GB40819	Venom allergen 3-like	3.62
GB40597	Bumetanide-sensitive sodium-(potassium)-chloride cotransporter	3.53
GB52724	Protein 5NUC-like	3.44
GB40820	Uncharacterized LOC100577824	3.43
***GB54806***	***Facilitated trehalose transporter Tret1-like***	3.39
GB50151	Odorant binding protein 9	3.35
GB45849	Clavesin-2	3.26
GB45658	T-box protein H15-like	3.18
GB54180	Protein gooseberry	3.10
GB52635	Pinin	3.10
GB51834	Sodium-dependent nutrient amino acid transporter 1-like	3.00
GB50806	Uncharacterized LOC413265	2.99
GB54418	Uncharacterized	2.89
GB54725	Cationic amino acid transporter 4	2.89

The “Average value” is the average of the normalized expression values of the three biological replicates for that tissue. Gene descriptions and gene identifiers in bold, italic font were further investigated using real time quantitative PCR (RT-qPCR).

**Fig 2:**
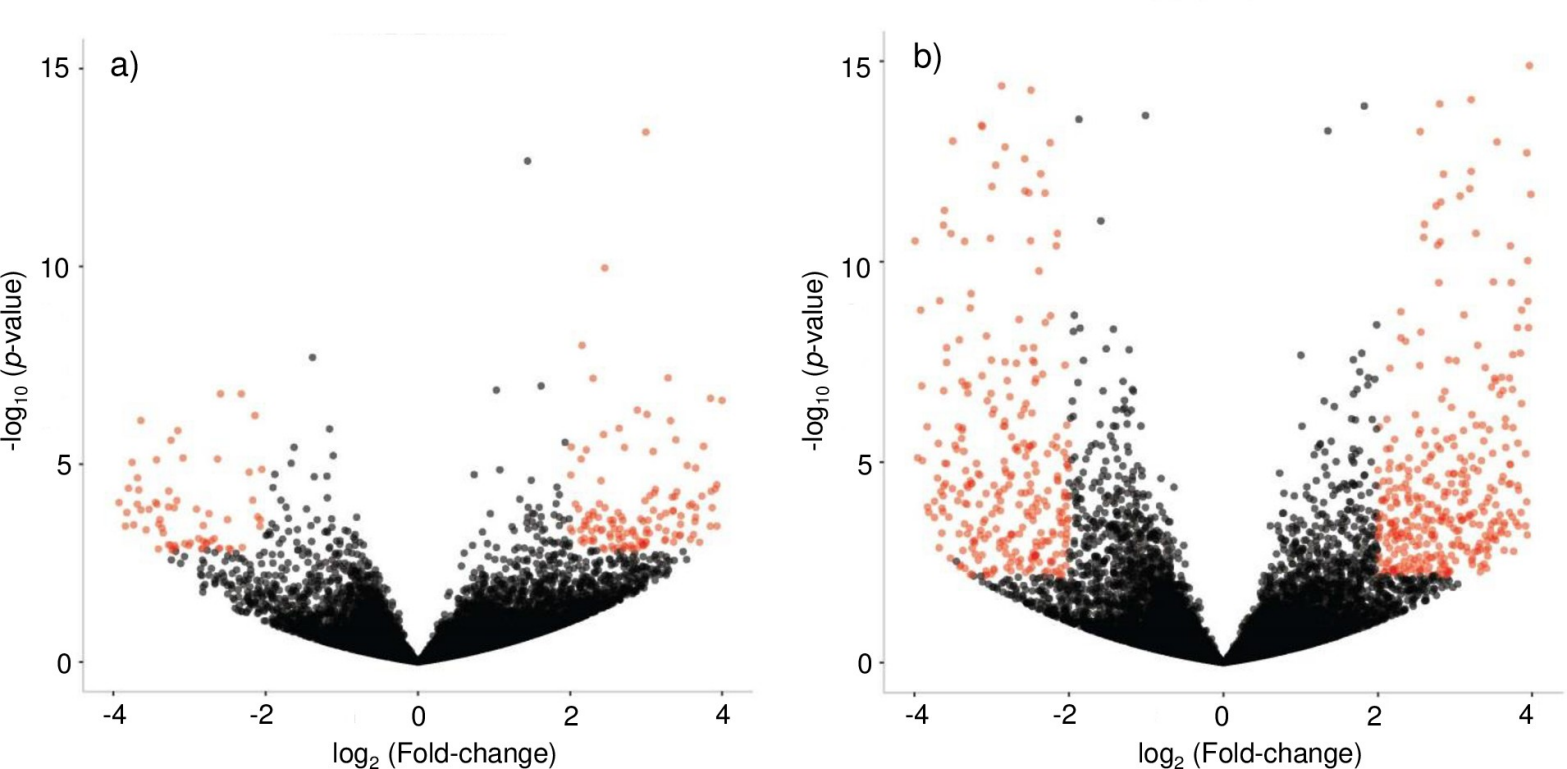
Volcano plots displaying differentially expressed genes (red-orange dots) between a) the spermatheca of mated and virgin honey bee queens, and b) the spermatheca of mated queens and drone semen. Each dot represents one gene. The black dots represent genes that were not differentially expressed (*P* < 0.01 and |log_2_ (Fold-change)| ≥ 2).

The expression levels of specific genes were compared between the three tissue types. Volcano plots (Fig 2 and S1 Fig) show individual genes plotted in comparisons of paired tissues, with the x-axis being log_2_ (Fold Change) and the y-axis being log_10_ (*P* value). The black dots represent individual genes with expression levels not significantly different between the two tissues (*P* > 0.01 and/or log_2_ (Fold Change) ≥ 2.0). The red-orange dots indicate genes with different expression levels between the two tissues (*P* < 0.01 and log_2_ (Fold Change) ≥ 2.0). In Fig 2A, red-orange dots on the left side of the volcano plot indicate genes that were down-regulated in mated queen spermatheca compared to virgin queen spermatheca, and red-orange dots on the right indicate genes that were up-regulated in mated queen vs. virgin queen spermathecae. Comparing that to the volcano plot with data from mated queen spermatheca and drone semen, the transcriptomes of the mated and virgin queen spermatheca are more similar than the transcriptomes of the mated queen spermatheca and drone semen (Fig 2B). The volcano plot comparing the transcriptomes of virgin queen spermathecae and drone semen (S1 Fig) is similar to the one shown in Fig 2B.

Comparisons of the RNA sequencing data from the three tissues revealed that the expression levels of 212 genes were significantly different between mated and virgin queen spermatheca (FDR < 0.05). A total of 129 genes (1.4% of total) were up-regulated and 83 genes (0.9% of total) were down-regulated in the mated queen spermathecae (S4 Table). Among these, the 20 genes with the greatest fold changes in expression between those tissues (ten highest and ten lowest) are shown in Table 2. In the genes that were up-regulated in the mated queen spermathecae, two gene products, “uncharacterized LOC102656393” (GB50654) and “uncharacterized protein PF11_0213-like” (GB41096), were also up-regulated in drone semen (S3 Table). This suggests that these uncharacterized protein mRNAs may be delivered to the spermatheca by sperm after mating. Other genes in Table 2 with high fold change values in mated vs. virgin queen spermatheca, such as “proline-rich extension-like protein EPR1” (GB40609) and “NF-kappa-B-inhibitor cactus-like” (GB53301), were also down-regulated in drone semen (S3 Table). The low expression levels of those genes in the virgin queen spermatheca and drone semen indicate that the expression of the genes is probably induced in the spermatheca after mating.

**Table 2:** The top ten and bottom ten genes that were differentially expressed between mated queen spermatheca and virgin honey bee queen spermatheca, ranked by the log_2_ Fold-change (LFC).

BeeBase gene identifier	Protein description	LFC	SEM	*p-*value	FDR
***Top ten up-regulated genes in mated queen spermatheca vs*. *virgin queen spermatheca***					
*** ***	*** ***	*** ***	*** ***	*** ***	*** ***
GB47485	Histone H2B	5.47	1.08	3.85E-07	0.00018
GB51032	Uncharacterized LOC100578048	4.01	0.87	3.95E-06	0.0010
GB51908	Adenylate kinase 8	4.00	1.06	0.000158	0.014
GB50654	Uncharacterized LOC102656393	4.00	0.77	2.43E-07	0.00014
GB46219	Nuclear factor interleukin-3-regulated protein	3.93	1.10	0.000366	0.023
GB50907	Uncharacterized LOC100577573	3.93	0.95	3.30E-05	0.0049
GB40609	Proline-rich extensin-like protein EPR1	3.91	0.95	4.22E-05	0.0060
GB53301	NF-kappa-B inhibitor cactus-like	3.85	0.95	4.86E-05	0.0065
GB41096	uncharacterized protein PF11_0213-like	3.85	0.74	2.15E-07	0.00013
GB43094	uncharacterized LOC726019	3.77	0.46	5.04E-16	5.4E-12
***Top ten down-regulated genes in mated queen spermatheca vs*. *virgin queen spermatheca***					
GB40136	Transmembrane protease serine 11B-like protein	-4.58	1.04	9.86E-06	0.0020
GB44100	Vegetative cell wall protein gp1-like	-4.60	1.07	1.60E-05	0.0028
GB44367	Phospholipase A2-like	-4.78	1.12	2.02E-05	0.0033
GB50477	Uncharacterized LOC100577527	-4.87	1.00	1.09E-06	0.00040
GB44295	Sodium-dependent neutral amino acid transporter B(0)AT3	-4.89	0.95	2.71E-07	0.00015
GB43248	***Alpha glucosidase 2***	-5.23	0.89	3.80E-09	6.3E-06
GB54549	***Alpha glucosidase***	-5.27	0.98	6.50E-08	5.6E-05
GB53978	Early nodulin-75-like	-5.28	0.96	4.11E-08	4.4E-05
GB53887	Uncharacterized LOC100578389	-5.83	0.95	1.02E-09	2.2E-06
GB44112	***Melittin***	-6.21	1.06	4.12E-09	6.3E-06

Genes are identified by their BeeBase gene identifier. SEM is the standard error of the mean of the log_2_ Fold-change. FDR is the False Discovery Rate. Gene names in bold font were used for confirmation of differential expression via RT-qPCR.

In comparing the RNA sequencing data from mated queen spermatheca and drone semen, 766 genes were differentially expressed: expression levels of 416 genes were greater and 350 genes were lower in the mated queen spermathecae (S5 Table). Table 3 shows the 20 gene products with the highest and lowest log_2_ fold changes. The 1,058 genes with differential expression levels in virgin queen spermatheca and drone semen are shown in S6 Table. Expression levels of 547 genes were greater in the virgin queen spermathecae and those of 511 genes were lower compared to drone semen.

**Table 3:** The top ten and bottom ten genes that were differentially expressed between mated queen spermatheca and drone semen, ranked by the Log_2_ Fold Change (LFC).

BeeBase gene identifier	Protein description	LFC	SEM	*p-*value	FDR
***Top ten up-regulated genes in mated queen spermatheca vs*. *drone semen***					
GB43575	***Trehalase***	7.56	0.92	#######	4.57E-14
GB47379	Histidine decarboxylase	7.34	0.76	#######	2.25E-19
GB49865	Protein drumstick	6.95	0.81	#######	2.76E-15
GB43576	Trehalase-like	6.60	0.96	#######	6.93E-10
GB55921	Esterase FE4-like	6.38	0.66	#######	3.66E-19
GB46537	Deoxyribonuclease-2-alpha	6.33	0.90	#######	2.92E-10
GB42557	Protein takeout-like	5.98	0.95	#######	2.64E-08
GB49751	Homeobox protein SIX3	5.94	0.89	#######	1.98E-09
GB55613	Uncharacterized LOC100576118	5.88	0.98	#######	1.22E-07
GB53925	Uncharacterized LOC724993	5.85	1.01	#######	4.67E-07
***Top ten down-regulated genes in mated queen spermatheca vs*. *drone semen***					
GB40598	***Bumetanide-sensitive sodium-(potassium)-chloride cotransporter***	-6.94	0.79	#######	3.55E-16
GB54180	Protein gooseberry	-7.08	0.74	#######	5.49E-19
GB52724	Protein 5NUC-like	-7.19	0.59	#######	1.54E-30
GB45850	***Clavesin-2***	-7.25	0.55	#######	7.91E-36
GB40819	Venom allergen 3-like	-7.25	1.08	#######	2.03E-09
GB54417	Farnesol dehydrogenase-like	-7.56	0.93	#######	1.14E-13
GB45658	T-box protein H15-like	-8.11	0.80	#######	3.48E-21
GB41857	CRISP/Allergen/PR-1-like	-8.39	1.03	#######	1.02E-13
GB48478	***Multiple inositol polyphosphate phosphatase 1***	-10.13	0.99	#######	1.48E-21
GB55813	Uncharacterized LOC100577773	-11.09	0.85	#######	4.62E-35

Genes are identified by their BeeBase gene identifier. SEM is the standard error of the mean of the log_2_ Fold-change. FDR is the False Discovery Rate. Gene names in bold font were used for confirmation of differential expression via quantitative RT-PCR.

### 3.3. Functional clustering analysis of differentially expressed genes

Gene contrasts that were up- or down-regulated by ≥ 4-fold in the transcriptomes of mated vs. virgin queen spermathecae were compiled in two different lists for D.A.V.I.D. functional annotation clustering analysis. In the list of 129 genes that were up-regulated in mated queen spermathecae, 73 genes were found in the D.A.V.I.D. database. From these 73 genes, a single cluster was identified belonging to the “DUF1676” category (domain of unknown function 1676) with an enrichment score of 1.83 (Table 4; protein descriptions are in S7 Table). In addition, 69 out of 83 genes in the list of genes that were down-regulated in mated compared to virgin queen spermathecae were found in the D.A.V.I.D. database. From these 69 genes, four different clusters were identified with enrichment scores ranging from 5.00 to 3.27 (Table 4; protein descriptions are in S7 Table). Many of these genes were categorized under “signal” or “hydrolase,” suggesting that signaling and metabolic pathways are affected in the spermatheca after mating.

**Table 4:** Cluster analysis of differentially expressed genes in mated queen spermatheca vs. virgin queen spermatheca using the online tool DAVID[50,51].

Annotation	FDR	Gene list
***Genes up-regulated in mated queen spermatheca vs*. *virgin queen spermatheca***		
***Cluster 1*: *Enrichment Score 1*.*83***		** **
Protein of unknown function DUF1676	0.0073	GB50561, GB50562, GB50572, GB50573, GB50568
***Genes down-regulated in mated queen spermatheca vs*. *virgin queen spermatheca***		
***Cluster 1*: *Enrichment Score 5*.*00***		
Signal	7.53E-10	GB44100, GB54485, GB50026, GB53911, GB50477, GB49796, GB42616, GB43248, GB44982, GB50116, GB55499, GB41760, GB44882, GB44058, GB41097, GB55408, GB42434, GB43739, GB46749, GB40092, GB52052, GB54549, GB45771, GB50761, GB44112, GB51733, GB50893, GB44367, GB49331, GB55388, GB49854
Hydrolase	1.41E-05	GB46749, GB47975, GB52052, GB50026, GB42616, GB50761, GB43248, GB46286, GB55499, GB41760, GB47299, GB41097, GB49854, GB43739
Glycoside hydrolase catalytic domain	0.0062	GB46749, GB43248, GB54549, GB42616, GB49854, GB42434
Glycoside hydrolase superfamily	0.018	GB46749, GB43248, GB54549, GB42616, GB49854, GB42434
***Cluster 2*: *Enrichment Score 3*.*72***		
Glycoside hydrolase catalytic domain	0.0062	GB46749, GB43248, GB54549, GB42616, GB49854, GB42434
Glycoside hydrolase superfamily	0.018	GB46749, GB43248, GB54549, GB42616, GB49854, GB42434
***Cluster 3*: *Enrichment Score 3*.*27***		
Hydrolase	1.41E-05	GB46749, GB47975, GB52052, GB50026, GB42616, GB50761, GB43248, GB46286, GB55499, GB41760, GB47299, GB41097, GB49854, GB43739
***Cluster 4*: *Enrichment Score 3*.*27***		
Hydrolase	3.19E-07	GB46749, GB47975, GB52052, GB50026, GB42616, GB50761, GB43248, GB46286, GB55499, GB41760, GB47299, GB41097, GB49854, GB43739

Recognized gene clusters were identified using the lowest stringency of the software's Functional Annotation Clustering settings. The reported genes are those that were clustered and had an FDR value < 0.05. Genes are reported using their BeeBase gene identifiers. Their protein descriptions are given in S7 Table.

Within the 416 genes that were up-regulated in mated queen spermathecae compared to levels in drone semen, 281 genes were found in the D.A.V.I.D. database. However, no functional clusters were identified with FDR < 0.05. Of the 350 genes that were down-regulated in mated queen spermathecae compared to drone semen, 222 genes were in the D.A.V.I.D. database. Within those, six different functional clusters of genes were identified with enrichment scores ranging from 5.94 to 2.22 (Table 5; protein descriptions are in S8 Table). Common categories of clusters were involved in membrane components such as transporters. Genes involved in membrane transport mechanisms were up-regulated in sperm but were less so in the spermatheca after mating.

**Table 5:** Cluster analysis of differentially expressed genes in mated queen spermatheca vs. drone semen using the online tool DAVID[50,51].

Annotation	FDR	Gene list
***Genes down-regulated in mated queen spermatheca vs*. *drone semen***		
***Cluster 1*: *Enrichment Score 5*.*94***		
Membrane	6.51E-05	GB50003, GB40392, GB54172, GB41034, GB54783, GB41033, GB54037, GB51046, GB46925, GB50001, GB43598, GB42062, GB41074, GB45210, GB54734, GB46119, GB53045, GB52634, GB43737, GB52775, GB41372, GB54145, GB51591, GB41129, GB45406, GB43584, GB50944, GB46663, GB53347, GB51598, GB42052, GB47662, GB49281, GB43180, GB54806, GB49259, GB44046, GB50128, GB46426, GB50232, GB50890, GB54578, GB49871, GB48643, GB42427, GB55881, GB43332, GB54311, GB45146, GB51566, GB50262, GB54610, GB51659, GB53427, GB53933, GB41313, GB41815, GB54298, GB45235, GB49227, GB54716, GB41670, GB49443, GB53792, GB49273, GB55095, GB52913, GB55505, GB48935, GB47876, GB43868, GB45393, GB51833, GB51834, GB42482
Transmembrane helix	6.68E-05	GB50003, GB40392, GB54172, GB41034, GB54783, GB41033, GB54037, GB51046, GB46925, GB50001, GB43598, GB42062, GB41074, GB45210, GB54734, GB46119, GB53045, GB52634, GB43737, GB52775, GB41372, GB54145, GB51591, GB41129, GB45406, GB43584, GB50944, GB46663, GB53347, GB51598, GB42052, GB47662, GB49281, GB43180, GB54806, GB49259, GB44046, GB50128, GB46426, GB50232, GB50890, GB54578, GB49871, GB48643, GB42427, GB55881, GB43332, GB54311, GB45146, GB51566, GB50262, GB54610, GB53427, GB51659, GB53933, GB41313, GB41815, GB54298, GB45235, GB49227, GB54716, GB41670, GB49443, GB53792, GB49273, GB55095, GB52913, GB55505, GB48935, GB47876, GB43868, GB51833, GB51834, GB42482
Transmembrane	7.15E-05	GB50003, GB40392, GB54172, GB41034, GB54783, GB41033, GB54037, GB51046, GB46925, GB50001, GB43598, GB42062, GB41074, GB45210, GB54734, GB46119, GB53045, GB52634, GB43737, GB52775, GB41372, GB54145, GB51591, GB41129, GB45406, GB43584, GB50944, GB46663, GB53347, GB51598, GB42052, GB47662, GB49281, GB43180, GB54806, GB49259, GB44046, GB50128, GB46426, GB50232, GB50890, GB54578, GB49871, GB48643, GB42427, GB55881, GB43332, GB54311, GB45146, GB51566, GB50262, GB54610, GB53427, GB51659, GB53933, GB41313, GB41815, GB54298, GB45235, GB49227, GB54716, GB41670, GB49443, GB53792, GB49273, GB55095, GB52913, GB55505, GB48935, GB47876, GB43868, GB51833, GB51834, GB42482
Major facilitator superfamily domain	0.0083	GB50003, GB41034, GB41033, GB54783, GB41670, GB4358,4 GB55881, GB52913, GB47662, GB54806, GB54610, GB50890, GB53933, GB43737
Transport	0.034	GB40392, GB54716, GB41033, GB45406, GB55881, GB53792, GB49273, GB50262, GB49259, GB55505, GB47876, GB50890, GB41313, GB43737, GB51833, GB42482, GB51834
Integral component of membrane	0.033	GB50003, GB40392, GB54172, GB41034, GB54783, GB41033 GB54037, GB51046, GB46925, GB50001, GB43598, GB42062, GB41074, GB45210, GB54734, GB53045, GB52634, GB43737, GB52775, GB41372, GB54145, GB51591, GB41129, GB45406, GB43584, GB50944, GB53347, GB46663, GB51598, GB42052, GB47662, GB49281, GB43180, GB54806, GB49259, GB44046, GB50128, GB46426, GB50232, GB50890, GB54578, GB49871, GB48643, GB42427, GB55881, GB43332, GB54311, GB45146, GB51566, GB50262, GB54610, GB53427, GB51659, GB53933, GB41313, GB41815, GB54298, GB45235, GB49227, GB54716, GB41670, GB49443, GB53792, GB49273, GB55095, GB52913, GB55505, GB48935, GB47876, GB43868, GB51833, GB51834, GB42482
***Cluster 2*: *Enrichment Score 2*.*77***		
Protein processing in endoplasmic reticulum	2.48E-04	GB41867, GB50944, GB51598, GB47336, GB56012, GB40866, GB51659, GB45210, GB43868, GB48812, GB48643, GB49871, GB55443
***Cluster 3*: *Enrichment Score 2*.*60***		
Major facilitator superfamily domain	0.0083	GB50003, GB41034, GB41033, GB54783, GB41670, GB43584, GB55881, GB52913, GB47662, GB54806, GB54610, GB50890, GB53933, GB43737
***Cluster 4*: *Enrichment Score 2*.*46***		
Major facilitator superfamily domain	0.0083	GB50003, GB41034, GB41033, GB54783, GB41670, GB43584, GB55881, GB52913, GB47662, GB54806, GB54610, GB50890, GB53933, GB43737
***Cluster 5*: *Enrichment Score 2*.*69***		
Transport	0.034	GB40392, GB54716, GB41033, GB45406, GB55881, GB53792, GB49273, GB50262, GB49259, GB55505, GB47876, GB50890, GB41313, GB43737, GB51833, GB42482, GB51834
***Cluster 6*: *Enrichment Score 2*.*22***		
Transport	0.034	GB40392, GB54716, GB41033, GB45406, GB55881, GB53792, GB49273, GB50262, GB49259, GB55505, GB47876, GB50890, GB41313, GB43737, GB51833, GB42482, GB51834

Recognized gene clusters were identified using the lowest stringency of the software's Functional Annotation Clustering settings. The reported genes are those that were clustered and had an FDR value < 0.05. Genes are reported using their BeeBase gene identifiers. Their protein descriptions are given in S8 Table.

### 3.4. Real time quantitative PCR (RT-qPCR) confirms RNA sequencing data

**Fig 3:**
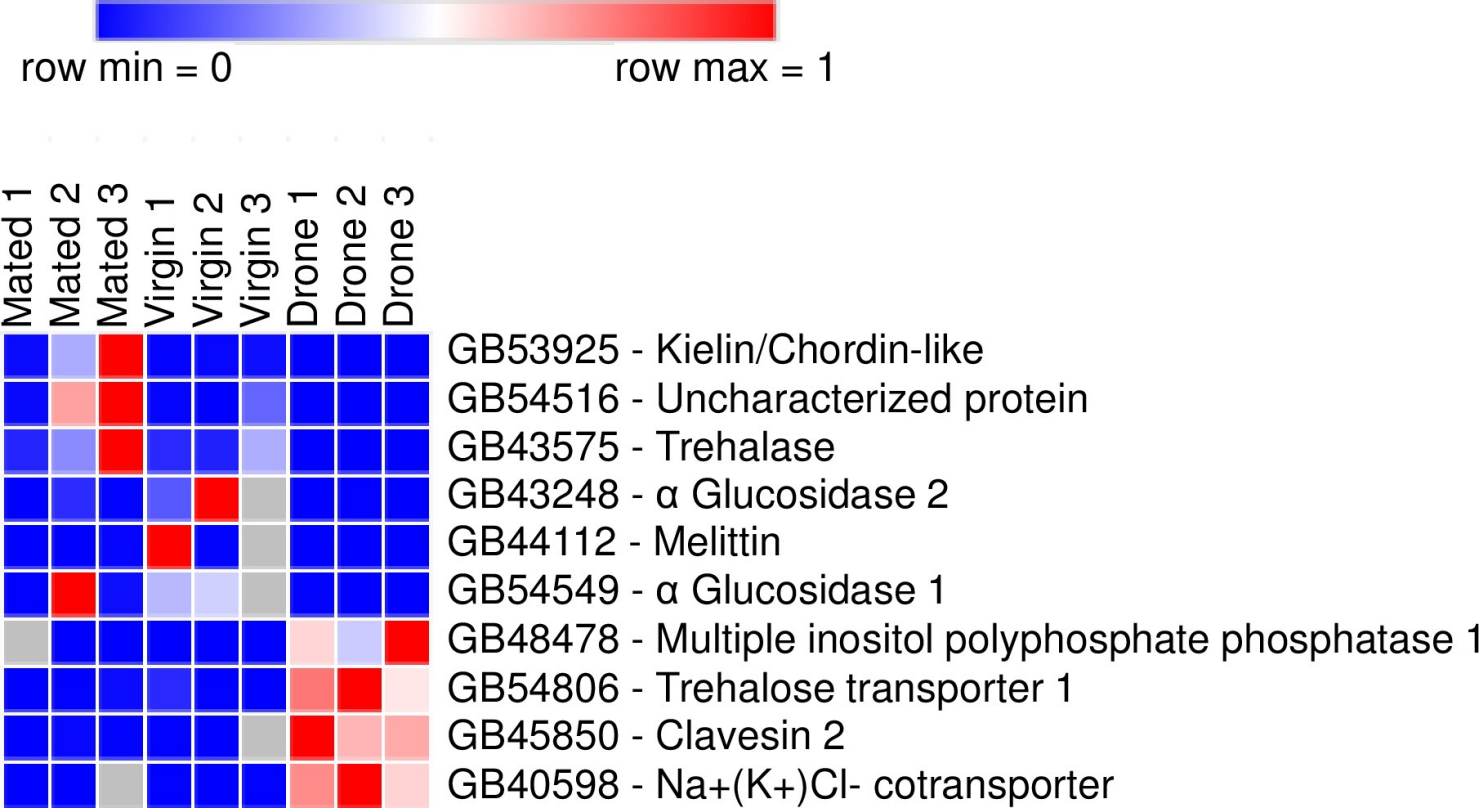
Heat map showing the relative expression levels of nine genes selected from RNA sequencing data for confirmation of differential expression by real time quantitative PCR (RT-qPCR). The genes are identified by their BeeBase gene identifier. The tissues used (n = 3 biological replicates per tissue type) were spermathecae from mated queens (“Mated”), spermathecae from virgin queens (“Virgin”) and drone semen (“Drone”). The color index above indicates genes that were expressed at relatively low levels (blue) or at high levels (red) in each row. Heat maps were generated using Morpheus (https://software.broadinstitute.org/morpheus/).

**Fig 4:**
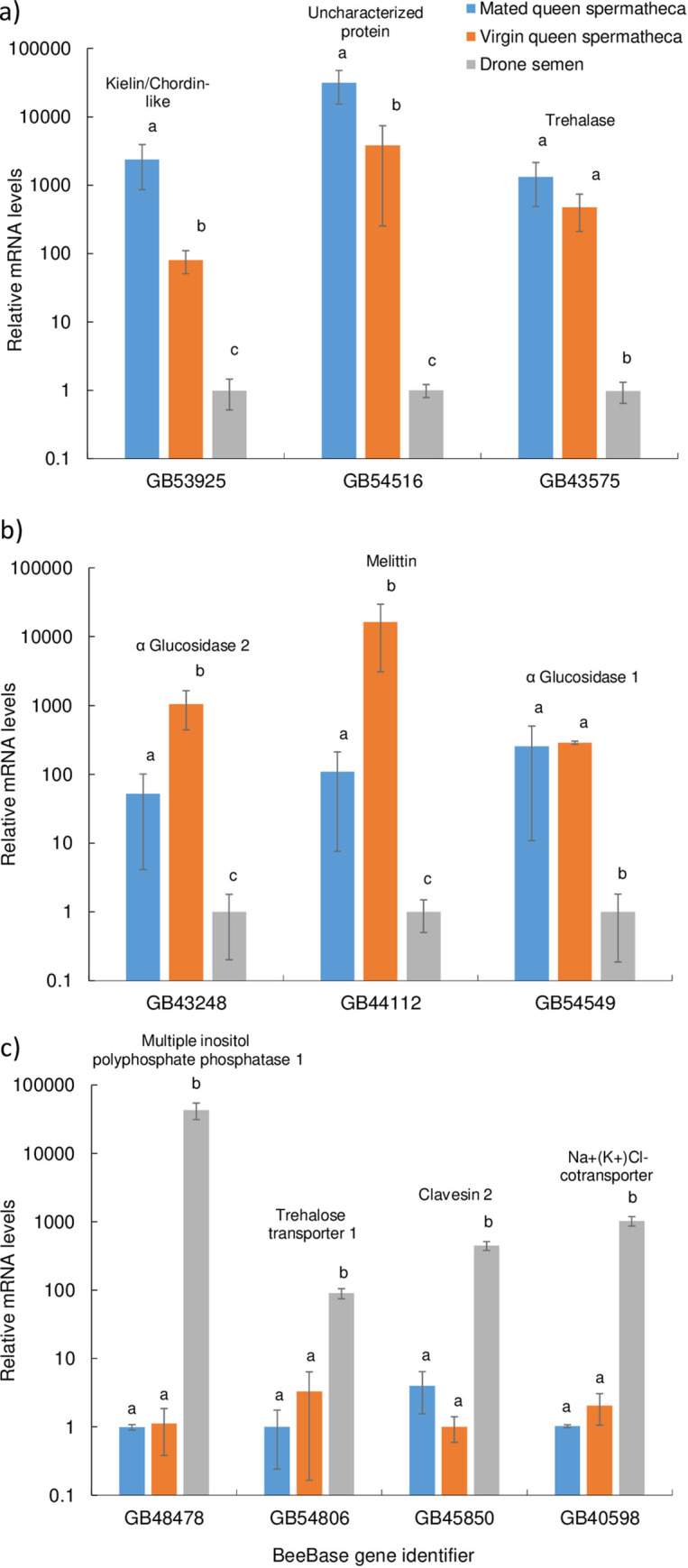
Real time quantitative PCR (RT-qPCR) confirmation revealed the relative expression levels of genes (identified by their BeeBase gene identifier) that were more highly expressed in mated queen spermatheca (blue bars), virgin queen spermatheca (orange bars), or drone semen (grey bars). Of the selected genes, a) two of three genes were most highly expressed in the mated queen spermatheca; b) two of three genes were most highly expressed in the virgin queen spermatheca; and c) four genes were most highly expressed in drone semen. Different letters above each bar represent the tissue for which gene expression was significantly different from that in the other tissues (*P* < 0.05). Linear values are presented after normalizing to the EF1aF2 mRNA. Protein names are listed above the bars.

Ten transcripts were selected for RT-qPCR analyses from RNA sequencing data (Fig 3), which revealed up-regulation of genes in one of the three tissues examined (spermatheca from mated queens, spermatheca from virgin queens and drone semen). The amplification efficiencies used for correction in all normalized fold-expression analyses ranged from 95.22% to 105.67%, which was within the 90–110% acceptable range [56]. The RT-qPCR analyses confirmed that two mRNAs (BeeBase gene identifiers GB53925 and GB54516, encoding kielin/chordin-like protein and an uncharacterized protein, respectively) were up-regulated in the spermathecae from mated queens when compared to the spermathecae from virgin queens or drone semen (Fig 4A). However, inter-animal variability precluded GB43575 mRNA (encoding Trehalase) from being differentially expressed between mated and virgin queen spermathecae samples. Furthermore, the GB43248 and GB44112 mRNAs (encoding α Glucosidase 2 and melittin, respectively) were up-regulated in the spermathecae of virgin queens compared to their expression in mated queens (Fig 4B). Levels of the GB54549 mRNA, encoding α Glucosidase 1, were similar between spermathecae from mated and virgin queens. All six gene products with RT-qPCR data reported in Fig 4A and 4B were up-regulated in queen spermathecae (regardless of mating status) compared to drone semen. In contrast, the four mRNAs (GB48478, GB54806, GB45850 and GB40598, encoding Multiple inositol polyphosphate phosphatase 1, Trehalose transporter 1, Clavesin 2, and Na^+^(K^+^)Cl^-^ cotransporter, respectively) were up-regulated in drone semen compared to queen spermathecae (Fig 4C). The RT-qPCR data confirmed some of the differences in mRNA levels first identified in the RNA sequencing data.

## 4. Discussion

In this study, RNA sequencing was used to provide a global, high throughput picture of the transcriptome of the spermathecae and semen of honey bees, as well as the relative expression levels of the genes. The purpose was to start to address the molecular mechanism through which honey bee queens store viable sperm for several years after mating. This is the first report of the complete transcriptome of the spermathecae of mated and virgin honey bee queens, in which over 10,000 genes were identified. This data complements a proteomic study of the spermathecal fluid of honey bee queens [34] in which, using one-dimensional acrylamide gel electrophoresis and mass spectroscopy, the authors identified 122 proteins in spermathecal fluid. Our study showed up-regulation of the vitellogenin (Vg) gene in mated queen spermatheca, which is involved in the production of the egg yolk protein necessary for egg production, as well as queen longevity [57]. This indicates that the presence of Vg as a major protein in that proteome may be translated in the spermathecae, as found the aforementioned study of the honey bee proteome [34].

Additionally, our current study provides the first full description of the transcriptome of semen from honey bee drones, with a similar number of expressed genes discovered as in the queen spermatheca. A previous study used the much less sensitive proteomic approach to identify 40 proteins in the sperm of honey bee drones [35]. The two proteomic studies [34,35] are consistent with our transcriptomic data, indicating that a distinct set of genes are expressed in honey bee spermathecae compared to those expressed in honey bee semen. A group studying queens of the social ant *Cremaster osakensis*, which also store sperm over several years, performed RNA sequencing of mated and virgin queen spermathecae [2]. They found 75 genes that were up-regulated in mated queen spermathecae compared to virgin queen spermathecae and 20 genes that were down-regulated in mated queen spermathecae. When compared to our discovery of 129 up-regulated and 83 down-regulated genes in the spermatheca of mated honey bee queens (S4 Table), both studies found approximately an eight-fold down-regulation of cytochrome P450 6a14, part of the cytochrome superfamily of genes involved in metabolism of xenobiotics and steroids [58]. In addition, both the ant and bee studies identified an eight-fold down-regulation of troponin C mRNA levels and 4-fold up-regulation of microtubule-associated protein futsch mRNA [59] levels in spermathecae in response to mating. Further studies are needed in these and other insect species to understand the molecular mechanisms required for long-term storage of sperm.

Many of the mRNAs that were up-regulated in drone semen (as obtained in our RNA sequencing and RT-qPCR results) were down-regulated in the mated queen spermatheca. This is because the amount of RNA contributed by the sperm in mated spermathecae is very low: only 0.5%. That is based on our RNA yields (note that they were very similar for virgin and mated spermathecae) and the fact that only 3% of total sperm deposited during mating (similar to our semen samples, pooled from ten drone ejaculates) make it to the spermatheca [12]. Another group similarly discovered changes in the levels of six selected mRNAs in ejaculated and stored sperm of honey bees [40]. For example, levels of ornithine aminotransferase mRNA were 10-fold greater in ejaculated sperm compared to sperm stored in the spermathecae for nine to 18 months. Therefore, it is likely that in the present study, in which mated queens only stored sperm for only up to four weeks before being dissected, we are viewing finite windows in gene expression that likely change over time during sperm storage in longer-lived queens. Moreover, we observed some inter-subject variability within biological samples for some of the treatments. For instance, there was variability of expression among mated queen tissues for the expression of the GB53925, GB54516 and GB43575 transcripts (see Fig 4). A larger number of biological samples could have aided in reducing the variability that is typically inherent across biological samples.

Despite some of these limitations, the transcripts chosen for quantitation by RT-qPCR included several genes that have been identified in other studies of honey bees and ants. For instance, some of these transcripts are involved in carbohydrate metabolism. Trehalose is the primary circulating sugar in insects. It is synthesized mainly by the fat body and its levels in hemolymph are regulated by its production and release by the fat body and its uptake by other body tissues [60]. The importance of the trehalose transporter (Tret1) in *A*. *mellifera* is highlighted by the fact that, in larvae, the molar ratio of trehalose to glucose in the hemolymph is 1.5 to 1. Cloning and expression of *A*. *mellifera* Tret1 mRNA in oocytes of *Xenopus* frogs allowed the high affinity of the trehalose transporter to be measured [60]. In larvae of moths in the genus *Bombyx*, the Tret1 gene is most highly expressed in testis, as measured by RT-qPCR [60], consistent with high levels in semen from honey bee drones (this study). There are much lower levels of Tret1 in other moth organs, including brain, abdomen, midgut, and ovary. In *C*. *osakensis* ant queens, levels of Tret1 mRNA were four times greater in spermathecae than in the whole body and they increased 2.2-fold in spermathecae within one week of mating [2]. Furthermore, *in situ* hybridization localized the Tret1 mRNA to the spermathecal gland and the hilar columnar epithelium of the spermathecal reservoir. In *Zootermopsis nevadensis* termite pre-soldiers, Tret1 mRNA levels are high in the head before molting [61]. In the current study of honey bees, we also identified greater levels of trehalase mRNA in spermathecae than in drone semen. Trehalase is an enzyme that circulates in the insect hemolymph and cleaves trehalose into two glucose molecules. Trehalase can be up-regulated by hormones (e.g., ecdysone) and pesticide exposure [62]. The observed up-regulation of the trehalose transporters in the mated queen spermatheca likely helps fulfill the organ’s energy requirements to maintain the sperm oxygenated and viable in the long term [60].

Two mRNAs encoding α glucosidases were also selected for RT-qPCR in this study because the RNA sequencing data indicated that they were up-regulated in the spermathecae from virgin queens compared to those of mated queens. Alpha-glucosidases are widely distributed in species ranging from plants to animals [63]. There are three α glucosidases (I, II, and III) in honey bees [64]. The α Glucosidase 1 protein is a predominant glucosidase in the ventriculus of honey bee workers [65], and cleaves both glucosides and maltooligosaccharides [66]. The α Glucosidase 2 protein is predominant in the ventriculus and hemolymph of honey bee workers [65], and it cleaves glucose residues in N-linked oligosaccharides. Moreover, real time semi-quantitative PCR data has previously shown that α Glucosidase 1 mRNA is more highly expressed in adult *Apis cerana* foragers than α Glucosidase 2 mRNA, while the levels of α Glucosidase 2 mRNA are greater in eggs, larvae and pupae [67]. Our RT-qPCR results confirmed that α Glucosidase 2 mRNA levels were greater in spermathecae from virgin honey bee queens compared to those in mated queens. The up-regulation of both α glucosidases in virgin queen spermathecae likely helps the organ break down polysaccharides for energy usage, especially when priming the sperm-storing organ in preparation for the energetic demands of storing and maintaining sperm viability over time.

Several of the other mRNAs analyzed by RT-qPCR encoded signaling molecules. The Multiple inositol polyphosphate phosphatase (Minpp1) enzyme cleaves inositol hexaphosphate (InsP6, also known as phytic acid) and the inositol pyrophosphates InsP7 and InsP8 [68]. These are ubiquitously expressed in eukaryotic cells and are believed to be master regulators of energy metabolism [69]. The amino acids of the Minpp1 enzyme of honey bees is only identical to human amino acid residues at 20 to 30% of the positions [68]. The Bumetanide-sensitive Na(K)Cl cotransporter is a membrane-bound ion channel. The Clavesin 2 protein regulates endocytosis and secretion [70]. The Kielin/chordin-like protein was recently recognized to be important for TGF1 beta signaling in mammals, as it binds Bone morphogenetic protein 7, increases binding to TGF type 1 receptors and interacts with activin A [71].

Previously, we reported up-regulation of three genes encoding antioxidant proteins in the spermathecae of mated queens compared to virgin queens [41]. In that study, RT-qPCR identified two-fold upregulation of the Catalase and Thioredoxin 2 genes along with three-fold upregulation of the Thioredoxin reductase 1 (TXNRD1) gene (*P* < 0.05). A different group found an increase in TXNRD1 mRNA levels in mated honey bee queen spermatheca compared to virgin queen spermatheca [34]. In this study, our minimal cutoff for the differential expression of genes was four-fold changes (log_2_ (Fold Change) ≥ 2.0; FDR < 0.05) in the level of gene expression. Our RNA sequencing data (S4 Table) confirmed the three-fold greater expression of the TXNRD1 gene in the mated spermatheca compared to the virgin queen spermatheca. Similarly, the RNA sequencing data in the current study determined that the expression levels of four other genes encoding antioxidants (Superoxide dismutase 1, Glutathione S-transferase D1, Glyoxalase domain-containing 4-like and Vitellogenin) were not different in the spermatheca of mated and virgin honey bee queens, consistent with the RT-qPCR data in our previous report [41]. In addition, in queens of the social ant *C*. *osakensis*, levels of Superoxide dismutase mRNA were not different between the spermatheca of mated and virgin queens [2].

In conclusion, this is the first known transcriptomic study that characterizes gene expression in the spermathecae of honey bee queens revealing the alterations in mRNA levels within them after mating. Further analysis of the honey bee queen transcriptomes obtained from this and other studies should help to identify genes that are involved in long-term sperm storage in the queen spermatheca after mating, and could potentially help to elucidate how environmental and biotic stressors can affect honey bee queen mating health.

## Supporting information

S1 Fig Volcano plot displaying differentially expressed genes (red-orange dots) between the spermatheca of virgin honey bee queens and drone semen. Each dot represents one gene. The black dots represent genes that were not differentially expressed (*P* < 0.01 and |log_2_ (Fold-change)| ≥ 2).
(PPTX)

S1 Table List of genes chosen for confirmation of differential expression by Real Time quantitiative PCR (RT-qPCR). An exception is the normalizer gene GB41358 at the bottom, on the last row. The BeeBase gene identifier, GenBank accession number, gene symbol, protein description, forward and reverse primer sequences, and amplicon size (bp) are provided.
(XLSX)

S2 Table RNA sequencing statistics for nine cDNA libraries. Each "Drone" replicate indicates a pooled semen sample from ten drones; each "Mated queen" replicate represents one spermatheca sample from a mated queen; and each "Virgin queen" replicate represents a spermatheca sample from a virgin queen. The "total number of reads mapped" is the number of reads that were mapped to the honey bee genome.
(XLSX)

S3 Table List of genes that were discovered during RNA sequencing. The BeeBase gene identifier, protein description, normalized gene values for each sample of the three tissue types analyzed, and the average normalized value for each tissue type, are provided. Each "Drone" replicate indicates a pooled semen sample from ten drones; each "Mated queen" replicate represents one spermatheca sample from a mated queen; and each "Virgin queen" replicate represents a spermatheca sample from a virgin queen. The genes were sorted (most prevalent to least prevalent) based on average values of the mated queen samples.
(XLSX)

S4 Table List of all genes that were differentially expressed between mated queen spermatheca and virgin queen spermatheca. The genes are ranked by the log_2_ Fold-change (LFC). Genes are identified by their BeeBase gene identifier. SEM is the standard error of the mean of the log_2_ Fold-change. FDR is the False Discovery Rate.
(XLSX)

S5 Table List of all genes that were differentially expressed between mated honey bee queen spermatheca and drone semen. The genes are ranked by the log_2_ Fold-change (LFC). Genes are identified by their BeeBase gene identifier. SEM is the standard error of the mean of the log_2_ Fold-change.
(XLSX)

S6 Table List of all genes that were differentially expressed between virgin honey bee queen spermatheca and drone semen. The genes are ranked by the log_2_ Fold-change (LFC). Genes are identified by their BeeBase gene identifier. SEM is the standard error of the mean of the log_2_ Fold-change.
(XLSX)

S7 Table Functional annotation clustering of differentially expressed genes in mated queen spermatheca vs. virgin queen spermatheca using the online tool DAVID [50,51]. Recognized gene clusters were identified using the lowest stringency of the software's Functional Annotation Clustering settings. The reported genes are those that were clustered and had an FDR value < 0.05. Genes are reported using their BeeBase gene identifiers and their protein descriptions. Genes in bold had their expression additionally examined by RT-qPCR. See S4 Table for a complete list of all the genes that were differentially expressed between mated queen spermatheca and virgin queen spermatheca.
(XLSX)

S8 Table Functional annotation clustering of differentially expressed genes in mated queen spermatheca vs. drone semen using the online tool DAVID [50,51]. Recognized gene clusters were identified using the lowest stringency of the software's Functional Annotation Clustering settings. The reported genes are those that were clustered and had an FDR value < 0.05. Genes are reported using their BeeBase gene identifiers and their protein descriptions. Genes in bold had their expression additionally examined by RT-qPCR. See S5 Table for a complete list of all the genes that were differentially expressed between mated honey bee queen spermatheca and drone semen.
(XLSX)
