# Supplementary material for: Transcriptomic analysis of the honey bee (Apis mellifera) queen spermathecae reveals genes that may be involved in sperm storage after mating
Source: PLoS One. 2021 Jan 8;16(1):e0244648. doi: 10.1371/journal.pone.0244648 (PMC7793254; doi:10.1371/journal.pone.0244648)
Supplement: S1 Fig — Each dot represents one gene. The black dots represent genes that were not differentially expressed (P < 0.01 and |log2 (Fold-change)| ≥ 2). (PPTX) [file pone.0244648.s001.pptx]

## Slide 1
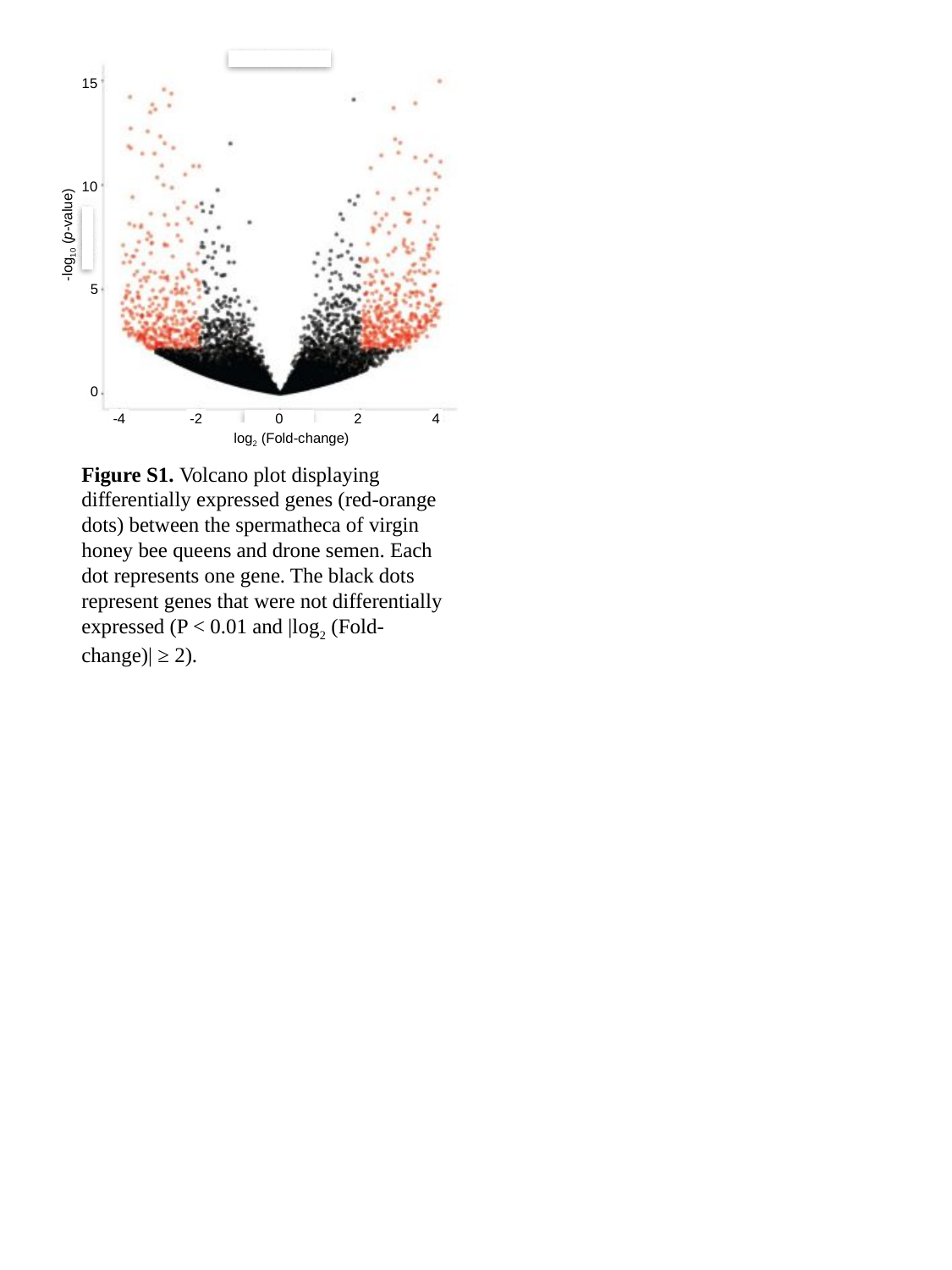

15
10
-log10 (p-value)
5
0
-4
-2
4
0
2
log2 (Fold-change)
Figure S1. Volcano plot displaying differentially expressed genes (red-orange dots) between the spermatheca of virgin honey bee queens and drone semen. Each dot represents one gene. The black dots represent genes that were not differentially expressed (P < 0.01 and |log2 (Fold-change)| ≥ 2).
